# Don't drop the patient: teamwork for cataract surgery

**Published:** 2014

**Authors:** Daksha Patel, Sally Crook

**Affiliations:** E-learning Director: International Centre for Eye Health, London School of Hygiene and Tropical Medicine, London, UK. Daksha.patel@lshtm.ac.uk; Programme Manager: Seeing is Believing, London, UK. Scrook@iapb.org

**Figure:**
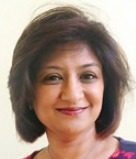
Daksha Patel

**Figure:**
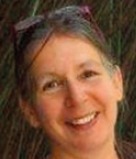
Sally Crook

The purpose of a team is to work together towards a common goal. On an athletics track, relay race teams run with a baton that is passed from one team member to another, without breaking the pace. If the baton is dropped, the team is disqualified.

The journey of the baton is a good analogy for the journey of someone who is visually impaired from cataract. Everyone has to work together within the health system to ensure that the patient is not ‘dropped’. In other words, the eye team must ensure that an informed patient, who is relaxed and mentally prepared for the next level of treatment, gets to a surgical centre and back home again.

The patient's journey begins when cataract is detected and the patient is informed about the possibility of treatment. This is done at the community level by health workers – such as primary health care workers and community health workers – who then refer the patient to an ophthalmic clinician for assessment and/or surgery. This stage of the journey has to be managed with sensitivity and reassurance for blind or vision-impaired patients, as they are likely to be afraid of surgery.

There is also a return journey: a cataract patient must come back to the community for follow-up and post-operative care, including detection of complications and referral back to the surgical centre if needed. This ‘return loop’ is important, both for the postoperative management of the patient and for the process of assessing outcomes (for quality control and auditing, and feedback to the surgical team).

**Low vision and rehabilitation**

Some patients may not achieve good vision after cataract surgery. This may be due to surgical problems, postoperative complications, or underlying eye diseases such as glaucoma. These patients may benefit from rehabilitation or low vision services, which range from learning to use assistive devices (e.g. magnifiers) to being taught new skills to improve employment opportunities. These services can maximise quality of life for people with vision impairments, and it is therefore important to include low vision or rehabilitation experts within your eye care team and to refer patients to them if needed.

**Figure:**
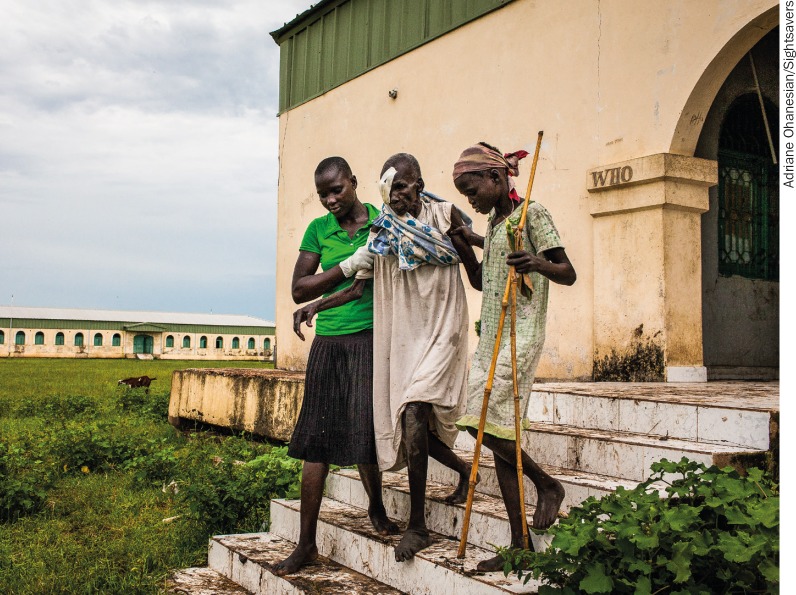
Everyone within the health system has to work together to ensure that a patient with cataract will receive the best possible care from start to finish

The patient's journey is completed only when the patient has been followed up and is satisfied with the services and the outcome. If not, then any issues must be addressed or resolved and constructive and helpful feedback should be given to the appropriate team members.

## Good working relationships – whose responsibility?

In order for health workers at the community level to successfully refer patients, there has to be a good working relationship – involving regular and pro-active communication, information sharing and feedback – between them and the team at the surgical centre.

We would like to suggest that much of the responsibility for good teamwork rests with the team at the surgical centre or eye unit, who rely on the health workers in the community to identify and refer patients for surgery and to care for them once they return to the community. The staff in the eye unit must consider and think about the staff delivering eye care in the community much, much more, and bring them into a bigger ‘eye team’. As an example, high-volume cataract surgical centres, such as those based in India, work closely with their referral networks to ensure uptake of surgery is not hindered due to delays in the pathways or at the point of treatment.

## Improving teamwork

There is much the team working at the eye unit can do to improve teamwork with their colleagues in the community.

### Strengthen communication

In order to successfully refer patients for surgery, there has to be good communication and information sharing between the workers at community level and the team at the surgical centre. Staff at the community/primary level should be confident that the information they are giving patients is correct and up to date.

New communication technologies, such as SMS (text messages), instant messaging (e.g. WhatsApp or BBM) and email have made it possible to be in immediate contact with colleagues out in the community. It may require some effort to gather everyone's contact details and set up the communications system. But once set up, such a system can be used to keep in regular contact with health workers in the community, provide them with feedback on successful (and unsuccessful) referrals, and inform them about any changes, for example if there is a change in the clinic dates or times. Both community and surgical centre workers should be trained in the communications system and motivated to use it. All of the above will help to ensure that workers in the community are not isolated or unsupported.

### Establish referral protocols

It is important to acknowledge the challenges patients face when they are referred for cataract surgery. The indirect costs of cataract surgery can be high and include transport costs and the lost wages of the patient and the accompanying person. It is therefore crucial that referrals are done carefully. If a patient arrives on a day when the clinic is closed or surgery is cancelled, they may not have the resources to come a second time.

A process for referral, using a protocol, would provide clear and consistent steps to follow. For example:

1. Explain to the patient what has been found and that their sight can be restored.
2. Tell the patient what to expect: what the treatment involves, its safety, possible outcomes and the benefits of treatment.
3. Ask about, and address, any fears or concerns the patient may have.
4. Inform the patient about where to go next, in enough detail (e.g. date, time, cost) to ensure that the patient can make the journey successfully and receive the care she or he needs.

Communication should be consistent and repeated as often as needed. Establishing a rapport with the patient – and understanding their concerns – will help with the acceptance of treatment. People working at community level who refer patients should have visited the surgical unit at least once, so they can tell the cataract patient exactly what to expect. There must also be a referral protocol back to the community so that people can be followed up and any complications managed after surgery.

Informing patients about postoperative complications – what to look out for and what they should do (including where they should go) – should be built into the post-operative protocol or referral pathway. One example is thickening of the posterior capsule, which is a common complication of cataract surgery. It involves gradual deterioration of vision and can be easily remedied. For patients, the visual improvement after surgery is always a remarkable experience. If vision deteriorates due to this complication, and patients don't know why, they could lose faith in the eye care system and not return for help, which is one way of ‘dropping’ the patient.

**Figure:**
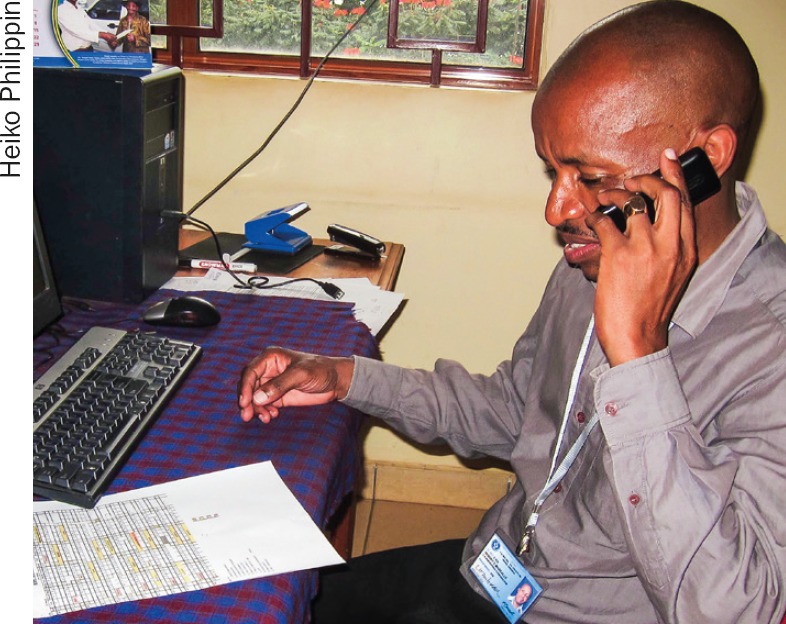
Regular communication and careful planning supports the patient's journey

### Offer support, supervision, and feedback

The important role played by health workers at community level is strengthened if they are provided with regular feedback about their performance by the district or secondary level team. Workers need to know who to ask for help with complicated and unusual cases, and must be given acknowledgement when they are doing well. Pre- and post-operative counselling of patients is essential and health workers should be supported with clear, consistent practice guidelines and information.

### Ensure reliable services

Surgical centres must establish a regular work plan or schedule that is shared with everyone at community level and updated regularly. Colleagues at community level must be informed of any changes or unexpected gaps in services (e.g. staff shortages) as quickly as possible (for example by SMS or via instant messaging) so that they can inform patients. Any staff shortages or service issues, such as broken down equipment or a shortage of consumables, should be urgently addressed.

### Practice good teamwork within the surgical eye unit

At the surgical centre, the manager must establish good information flow between team members within the eye unit and between them and the health workers in the community. Recognising the various roles within the team (including finance, administration, reception, stores, equipment maintenance, cleaning and pharmacy) will also help to ensure the smooth functioning of the unit. The result – regular procurement of medicines and instruments, good patient flow into theatre and aseptic functioning in operating theaters – will boost both the number of operations and their quality.

Good working relationships between the team at the eye unit and the health workers in the community can lead to more effective service delivery, as steps can be taken to ensure neither group is overburdened or under-used. Regular communication (ideally between named individuals) and clear, well-understood protocols will provide a framework for daily interactions.

Teamwork can be further strengthened by training, mentoring and active support for workers in the community. All of the above will ensure that the patient receives timely and high-quality service in an efficient and user-friendly style.

**Time to reflect**

Do you have the following in place?

1. A registration system that links different services (e.g. refractive services or the diabetes clinic) to the eye department and keeps patient records together.
2. Agreed referral protocols, including referral to low vision and rehabilitation services.
3. An arrangement for workers at community level to observe-cataract surgery so they can inform patients about the procedure and about the hospital.
4. Pre-operative counselling at community level to address the fears of people who are older, blind or vision impaired.
5. Post-operative follow-up and reassurance for the patient, and information about the possibility of complications (including what to look out for, what to do, and where to go).
6. Communication pathways, both within the eye unit and out to health workers in the community. This includes having the names and contact details of key people within the hospital, and arranging regular opportunities for the team at the surgical centre to meet the health workers who form part of their referral network in the community.
7. Good inventory management and procurement protocols.
8. Human resource mechanisms to ensure that posts are filled and that people are in place to do the work of those who are on leave.
9. A system for recording patient satisfaction, taking any needed action, and giving feedback to staff.

